# Perceptions of dolutegravir-based regimen use among people living with HIV in Limpopo province: A qualitative study

**DOI:** 10.4102/curationis.v49i1.2815

**Published:** 2026-02-20

**Authors:** Zandile R. Sibeko, Boitumelo J. Molato, Salaminah S. Moloko-Phiri

**Affiliations:** 1 NuMIQ Focus Area, School of Nursing Science, Faculty of Health Sciences, North-West University, Mahikeng, South Africa; 2 Department of Nursing, Faculty of Science, Agriculture and Engineering, University of Zululand, KwaDlangezwa, South Africa

**Keywords:** dolutegravir, HIV, perception, people living with HIV, antiretroviral therapy

## Abstract

**Background:**

HIV and AIDS continues to be a critical public health challenge, with sub-Saharan Africa shouldering the heaviest burden. Progress towards disease management has been implemented over the years, and antiretroviral therapy (ART) has been the most significant milestone. The World Health Organization recommended dolutegravir as the second line of treatment for the disease. A dolutegravir-based regimen has been appreciated for its great benefits. While its effectiveness is apparent, reports of side effects have raised concerns about adherence, posing a challenge to long-term treatment success.

**Objectives:**

This study explored the perceptions of people living with HIV (PLHIV) in Limpopo province regarding the use of dolutegravir-based regimens.

**Method:**

A qualitative, explorative-descriptive design was followed. Twenty individual semi-structured interviews were conducted. Purposive sampling and sample size were determined when data saturation was reached. Thematic analysis was used.

**Results:**

Four major themes emerged: high acceptance of dolutegravir, limited knowledge of treatment details, the burden of managing long-term ART and barriers to adherence, including concerns about side effects.

**Conclusion:**

While dolutegravir was generally well accepted, there were notable deficiencies in understanding treatment, underscoring the importance of improving patient education to ensure enhanced adherence to treatment. This study provides critical insights into the lived experiences of PLHIV using dolutegravir, offering valuable direction for future interventions aimed at improving adherence and optimising treatment outcomes in HIV care.

**Contribution:**

The study provides context-specific insights into how PLHIV perceive and experience dolutegravir-based regimens. By uncovering both the drivers of acceptance and the hidden barriers to adherence, it offers actionable evidence to guide patient-centred ART counselling, inform policy decisions and strengthen HIV treatment programmes to achieve sustained viral suppression in similar settings.

## Introduction

The human immunodeficiency virus (HIV) and acquired immunodeficiency syndrome (AIDS) continue to pose significant challenges to global public health, especially in sub-Saharan Africa, which experiences the greatest burden of disease. Since the initial identification of HIV cases in the early 1980s, first noted among men who have sex with men, the epidemic has developed into a substantial threat to human health and survival (Mayer et al. 2021). Without treatment, HIV progressively weakens the immune system by attacking CD4 cells, leaving individuals vulnerable to opportunistic infections and life-threatening conditions.

Over the past four decades, remarkable scientific advances have transformed HIV from a fatal illness into a manageable chronic condition. The scale-up of antiretroviral therapy (ART) has been the most significant milestone, dramatically reducing AIDS-related morbidity and mortality worldwide. By the end of 2021, an estimated 34 million people were living with HIV (PLHIV), with approximately 24.5 million accessing ART (Wembulua et al. 2024). Nonetheless, the epidemic remains unevenly distributed, with Eastern and Southern Africa accounting for more than 23 million cases. South Africa, the epicentre of the epidemic, has an estimated 7.8 million PLHIV representing 18.7% of the population aged 15 to 49 years (Zakumumpa et al. 2021).

Despite significant advancements, challenges continue to exist in maintaining long-term treatment efficacy, enhancing adherence and managing drug-related adverse effects within the HIV management cascade. Continuous research and innovation have resulted in the emergence of new ART alternatives, such as dolutegravir (DTG), which the World Health Organization (WHO) has endorsed because of its effectiveness, tolerability and substantial genetic resistance barrier (Temereanca & Ruta 2023). Alongside these advancements, ongoing evaluation of patient experiences with new regimens has been essential to ensure that treatment strategies remain effective, acceptable and sustainable.

### Background

In recent years, the WHO has prioritised the advancement of ARTs that are safer, more tolerable and highly effective (Cohn et al. 2015; WHO 2022). In 2013, the WHO recommended a fixed-dose combination regimen including efavirenz (EFV) as the first-line treatment for PLHIV. While EFV was widely adopted, it has been criticised for adverse side effects and a low genetic barrier to resistance (Calmy et al. 2020). These limitations present challenges to achieving Sustainable Development Goal (SDG) 3.3 and the UNAIDS 95-95-95 targets aimed at ending AIDS as a public health threat by 2030.

Over the past 25 years, there has been remarkable progress in various types of ART, which has significantly improved treatment outcomes for PLHIV. This progress has ushered in the era of highly active antiretroviral therapy (HAART), where three different agents work together to block viral replication (Ifedilichukwu & Joy 2023). Nonetheless, the challenge of achieving long-term viral suppression while minimising toxicity remains a key goal. In response to these challenges, the WHO has recommended the use of DTG-based regimens. These are usually paired with tenofovir (TDF), lamivudine (3TC) and DTG or with abacavir (ABC), 3TC and DTG (Lee et al. 2023). These regimens are recommended both for treatment-naïve patients and for those with prior ART experience. This includes adults and children aged 12 years and older who weigh at least 40 kg. This study focused specifically on the fixed-dose combination of TDF, 3TC and DTG.

Dolutegravir belongs to the integrase strand transfer inhibitor (INSTI) class and is favoured for its potency, safety profile and high barrier to resistance (Henegar et al. 2023). By mid-2019, the rollout of DTG-based treatment plans was already in progress in several low- and middle-income countries, including Uganda, Botswana and South Africa, but it was not until 2 years later that widespread implementation occurred (Bacha et al. 2023). Studies have shown that DTG-based regimen can efficiently lower viral loads rapidly, but there is still limited understanding of how PLHIV perceive and experience this treatment (Cahn et al. 2022; Casalini et al. 2023; Mathur 2023). In sub-Saharan Africa, DTG-based regimen research has concentrated on specific groups, like pregnant women or particular communities, which leaves a significant gap in our understanding of how the broader population views these regimens (Chapola et al. 2023; Rasi 2021). There is a clear need for more extensive research that investigates the perceptions of a broader population.

Emerging qualitative evidence from various settings highlights both positive and cautious perceptions of dolutegravir among PLHIV. Studies from several African contexts, including Uganda, Malawi, Nigeria and Kenya, reported that patients tend to have positive perceptions about the DTG-based regimen because of significantly faster viral suppression and fewer side effects (Chapola et al. 2023; Eze et al. 2023; Nalugga et al. 2022; Alhassan 2020). Nevertheless, concerns about insomnia, weight gain and uncertainty around safety, especially for women of reproductive age, were reported. The uncertainty and hesitancy about the DTG-based regimen’s effects on fertility were contributed to by limited support or counselling. This led to an ongoing misconception that raised concerns that were avoidable (Massawe et al. 2023). In India, similar mixed emotions were noted among participants, with study participants indicating overall appreciation of the DTG-based regimen’s effectiveness, while indicating it caused issues related to sleep and weight (Mathur 2023). Collectively, these findings suggest that, while there is an overall conception of the DTG-based regimen being accepted as a contemporary and effective therapy for managing HIV, patient experiences remain shaped by individual health responses, gender considerations and the degree of quality provided in health intervention information. This highlights the need for more context-specific research to delve deeper into this issue.

Despite all that, the WHO has recommended caution when prescribing DTG to pregnant women because of a potential increase in the risk of neural tube defects (Todorović, Dragović & Lukić 2024). In addition, patients with drug-sensitive tuberculosis (DS-TB), using DTG, require careful oversight as rifampicin can lower DTG plasma levels (De Nicolò et al. 2022). While adjusting the dosage can help manage this interaction, the limited availability of the drug in resource-limited settings can make consistent use a challenge (John 2024).

Given these factors, there’s an ongoing discussion about whether the advantages of the DTG-based regimen outweigh its potential downsides. The existing research has extensively concentrated on clinical effectiveness, with limited focus on the experiences of patients using the treatment itself. It is crucial to understand how patients perceive their treatment, as this could help identify what influences their adherence, shape counselling approaches and inform policy decisions. This understanding is particularly vital in South Africa, where the rates of HIV are among the highest in the world. Therefore, this study aimed to explore and describe the perceptions and lived experiences of PLHIV regarding the use of a DTG-based regimen in Limpopo province.

### Problem statement

In Limpopo province, the transition to DTG-based regimens has been rapidly implemented as part of South Africa’s ART programme (Hanser 2021; Ramusilei 2023). The clinical experiences indicate that many patients are embracing this change and the acceptability of the new regimen; however, there have been reports of side effects such as rashes, nausea, vomiting and heavy menstrual bleeding that have caused some individuals to discontinue their treatment. These discontinuities risk undermining viral suppression efforts and reversing gains made in HIV care. There is a lack of empirical evidence on how PLHIV in Limpopo perceive the DTG-based regimen, what factors influence their acceptance or rejection of this treatment and how these views affect their adherence. Without this insight, any strategies aimed at addressing patient concerns and promoting the ongoing use of DTG might fall short. This study aims to fill that gap by examining and exploring perceptions of PLHIV regarding the use of DTG-based regimens in Limpopo province.

### Purpose of the study

The purpose of this study was to explore the perceptions and lived experiences of PLHIV in Limpopo province regarding the use of DTG-based regimens in order to inform strategies for improving patient-centred HIV care.

### Contribution to the field

This study aimed to shed light on context-specific evidence regarding perceptions, acceptance and concerns of PLHIV about DTG-based regimens in Limpopo province. The insights from patients about their experiences and the factors that affect their adherence will help shape targeted interventions to address the management of side effects, enhance treatment acceptability and maintain high viral suppression rates. In addition, the results will serve as a valuable tool for policymakers, healthcare providers and programme managers, helping them refine ART counselling, manage side effects and develop better patient support strategies to improve HIV treatment outcomes in similar resource-limited and rural areas.

## Research methods and design

### Study design

This study aimed to explore the perceptions and lived experiences of PLHIV regarding the use of DTG-based regimens. Qualitative research is essential in understanding behavioural science, particularly when investigating the motivations and factors influencing human behaviour (Anas & Ishaq 2022). In this research, a qualitative, exploratory-descriptive and contextual approach was utilised to better comprehend the perspectives of PLHIV regarding the use of the DTG-based regimen. The exploratory design allowed the researcher to explore participants’ experiences and viewpoints and gain valuable insights into their perceptions of DTG-based regimen use. The descriptive aspect of this method aided participants in expressing their thoughts and lived experiences with the regimen. Lastly, the contextual design added significance as the research was conducted in clinics where participants receive ART. This ensured that the findings were rooted in the real-world settings of their treatment environment. This approach gave the researcher a complete picture of how patients view things in their everyday lives.

### Study setting

The research was conducted in Limpopo province, focusing on selected clinics within the Ephraim Mogale sub-district of the Sekhukhune District. The sub-district had a total of 10 primary healthcare clinics that provide HIV services. A systematic sampling was employed, and all 10 clinics were arranged alphabetically, and every third clinic was selected, starting with the first on the list. This procedure resulted in the inclusion of three clinics. This sampling approach ensured that the total number of clinics in the sub-district guided the sample size for the clinics. This promoted a balanced and fair representation across the area, with the feasibility required for in-depth qualitative data collection and equal opportunity for selection across clinics. Despite the small sampling frame, the systematic approach provided a transparent and unbiased means of site selection suitable for the qualitative nature of the study.

### Study population and sampling strategy

A population is a group of individuals that the researcher intends to study. Sampling is selecting a representative sample of individuals from the population of interest (Willie 2024). The study population consisted of PLHIV, who were males and females aged 18 years and older, who had been on a DTG-based regimen for at least 6 months and were willing to participate. A non-probability purposive sampling approach was used to recruit participants who could provide rich, relevant and diverse perspectives on the use of DTG-based regimens and have the characteristics to fulfil the study’s objectives. Given the topic’s sensitivity, the researcher also used convenience sampling, recruiting patients already present at the selected clinics during the recruitment process. Twenty participants were interviewed, and data collection continued until saturation was reached.

### Data collection

Data collection is gathering and measuring information on the variables of interest being studied (Depoy 2024). The first author, who was also a student researcher, will be referred to as the ‘researcher’ throughout this manuscript. Authors two and three were supervisors. The researcher was responsible for conducting all stages of the research process, including data collection and engagement with the study sites. The researcher reached out to the Sekhukhune Department of Health in Limpopo 1 week prior to the planned data collection date to obtain permission for access to the selected clinics. In addition, in-person visits were made to the Ephraim Mogale sub-district area managers and the operational managers of the selected primary healthcare clinics. During these visits, the researcher provided a brief study overview and presented all necessary approval letters from the university and the department of health, the national office that authorised conducting the study.

Semi-structured individual interviews were then conducted over a period of 6 days at the three selected sites in Ephraim Mogale sub-district, with 2 days spent at each clinic. Before each interview, informed consent was obtained by a research assistant. These 6 days effectively gathered rich data about the perceptions of PLHIV regarding the use of a DTG-based regimen. The researcher facilitated the interviews using an interview guide that included open-ended questions, with the primary question being, ‘What are the perceptions of PLHIV regarding the use of the dolutegravir regimen in Limpopo province?’ This was followed by probing questions. The interview guide was designed to address the study’s objectives. It was provided in Sepedi, which is the widely spoken language, and English to accommodate those who do not speak Sepedi.

Each interview lasted approximately 30 min; however, the duration of the interviews solely depended on the participant’s willingness and knowledge to engage in the topic studied. Audio recordings were made, but participants’ permission was asked prior to commencing the recording. Nonverbal cues and body language were also observed during interview sessions. The researcher transcribed the data, which were subsequently reviewed by supervisors experienced in qualitative research to ensure transcription accuracy. Data saturation, the point at which no new information emerged from the interviews, was reached after interviewing the 13th participant; however, seven additional participants were interviewed to confirm saturation.

### Data analysis

Qualitative data analysis involves organising and making sense of the data collected (Depoy 2024). The authors also stated that researchers make sense of the collected data by identifying patterns, themes, categories and regularities. Thematic data analysis steps were used to analyse data. The analysis was done by reading each transcription to obtain its main idea. All 20 transcripts were analysed, and similarities were grouped to formulate themes and sub-themes. The principal researcher conducted the analysis. An independent co-coder experienced in qualitative research was sent the transcripts to verify the coding themes. Both the researcher and the co-coder agreed on the same themes. A meeting with the supervisors was held to compare the themes and the final themes, as a consensus was reached.

Thematic analysis was employed using Braun and Clarke’s six-step framework, as cited by Ahmed et al. 2025. The six steps are as follows: (1) becoming familiar with the data, (2) generating initial codes, (3) searching for themes, (4) reviewing the themes, (5) defining themes and lastly (6) writing up final themes. The researcher evaluated every transcription to discover key concepts, categorising commonalities into themes and sub-themes. To ensure rigour, a skilled co-coder conducted an independent analysis of the transcripts, and both individuals (the researcher and the co-coder) agreed on the final themes. Supervisors additionally examined the themes to validate the analysis and enhance trustworthiness.

### Trustworthiness

Trustworthiness is an essential aspect of any research study, and the authors defined trustworthiness as the researcher’s confidence in the study’s accuracy and truth (Depoy 2024 trustworthiness, the study would be of little value. Therefore, principles of credibility, transferability, dependability and authenticity were implemented in this study to maintain trustworthiness.

Credibility is the degree to which the research represents the actual voice of the research participants (Flanagan & Beck 2024). The researcher engaged in prolonged interactions during the interview sessions to ensure credibility, allowing for a deeper understanding of the participants’ realities. Prolonged engagement with participants allowed the researcher to probe for more in-depth narratives regarding their perceptions of the dolutegravir-based regimen in Limpopo province. The findings of the study are reliable and trustworthy. The research was conducted meticulously, with careful collection and analysis of the data. The conclusions drawn are supported by evidence from the literature, instilling confidence in the validity of the findings.

Transferability determines if the study’s findings can be applicable from one specific situation to another (Enworo 2023). For transferability purposes, the researcher developed a detailed research method, and a thick description of the participants’ perceptions regarding the dolutegravir-based regimen was outlined so that the reader could transfer the study’s findings to other settings.

Dependability refers to the ability of the findings to show consistency and repeatability (Enworo 2023). The researcher ensured dependability by repeatedly listening to the audio recordings and re-reading the transcripts to verify the accuracy of the identified themes and sub-themes. Additionally, the audio recordings and transcripts were submitted to the co-coder to confirm that the formulated codes accurately reflected the participants’ perceptions of using the dolutegravir-based regimen. The findings of this study align with previous research, indicating that a similar analysis could yield comparable results in different settings.

Confirmability is the degree to which the participants shape the findings of a study and not the researcher’s bias, motivation or interest (Enworo 2023). To ensure confirmability, the researcher remained neutral and unbiased during the execution of the study. Triangulation was used to check for consistency of the findings generated to ensure that they account for rich, robust and comprehensive findings of the lived experience of PLHIV regarding the use of dolutegravir-based regimens in Limpopo province.

### Ethical considerations

Ethical clearance to conduct this study was obtained from the North-West University Health Research Ethics Committee (NWU-HREC) on 5 May 2022. The ethics approval number is NWU-00021-22-S1. This study was conducted in accordance with the ethical principles outlined in the Declaration of Helsinki. This study used the NWU standardised consent form. The research question was used to formulate the interview schedule. The study involved human participants, and animals or human tissues were not applicable. Zandile R. Sibeko, who was a student at the time, undertook the study only after all necessary written ethical approval was formally granted. Prior to participation, all participants were fully informed about the study’s purpose, procedures, potential risks and their right to withdraw at any time without penalty. As all participants provided written consent, no alternative consent methods were employed. Approval to access the selected clinics was also obtained from the Limpopo Department of Health and the relevant district office. Their personal information was kept private, with any identifying details left out of the findings to maintain confidentiality. The participants were thoroughly briefed on the disclosure of the study results and their rights to confidentiality. The interviews took place in a private room that was not exposed to external disturbances.

## Results

### Demographic profile

The results are discussed in this section. Twenty participants took part in the study (see Table 1). The majority of participants were treatment-experienced patients. No gender was dominant in this study. The age group of participants was 30 years – 39 years.

**TABLE 1:** Demographic characteristics of the participants (*N* = 20).

Characteristics	Variables	*n*
Gender	Female	10
	Male	10
Age (years)	18-29	4
	30-39	12
	40-45	4
Marital status	Single	16
	Married	2
	Not married but staying with partner	2
Level of Education	No schooling	0
	Primary school	2
	Secondary school	3
	Matric	15
	Tertiary	0
Employment	Employed	15
	Unemployed	5
	Pensioner	0

Table 2 describes the themes and sub-themes that emerged through participants’ responses on their perceptions of using a DTG-based regimen.

**TABLE 2:** A summary of the themes and sub-themes.

Themes	Sub-themes
1. Acceptance of new treatment	1.1. Benefits of new treatment, dolutegravir 1.2. Persistent adherence to new treatment dolutegravir
2. Level of knowledge regarding the new treatment (dolutegravir)	2.1. Insight into the importance of taking new treatment 2.2. Lack of knowledge regarding the new treatment (dolutegravir)
3. The burden of taking the previous regimen	3.1. Side effects of the previous regimen 3.2. Side effects of DTG-based regimen
4. Reasons for non-adherence to dolutegravir	4.1. Alcohol use and work-related issues

ART, antiretroviral therapy; DTG, dolutegravir.

The following themes and related sub-themes were identified in the study: (1) acceptance of new treatment, (2) level of knowledge regarding the new treatment of dolutegravir, (3) the burden of taking ART treatment, and (4) reasons for non-adherence to dolutegravir.

#### Theme 1: Acceptance of new treatment

Acceptance of the new treatment, dolutegravir, emerged as the first theme, and the benefits of the new treatment and persistent adherence to the new treatment were identified as the sub-themes.

**Sub-theme 1.1: Benefits of new treatment, dolutegravir:** The following reflections from most participants indicated that the new treatment known as dolutegravir had acceptable benefits and that their lives had changed as they felt better. A standard view was that the new treatment did not inflict any problems in their lives. It was also indicated that dolutegravir enhanced strength, energy and appetite stimulation. These were expressed as follows:

‘Uhmm, according to me, the medication treats me well and does not give me problems; it is just okay. I’m not sure if it’s because I am used to them or what, but according to me, they are very good to me. No, this medication is suitable; I said they started treating me well initially. When they changed me to these, I thought I would experience side effects like my previous treatment until I got used to them, but when I took them at night, I did not have any problem. The following day, I had no problem again, and then I figured these were better than my previous ones.’ (Participant A, Male, 36 years)

‘I never had any problem from the word go with this treatment until now; even my child was born fine. My child was born HIV-negative. I think it’s because of the treatment, and I am taking it correctly. My wife is also HIV positive, but we live normally. The thing is, we were told at the clinic that if we take our treatment well, we will give birth to an HIV-negative child. So, we did that because we wanted our child to be clean.’ (Participant G, Male, 40 years)

‘Aaah. My body also changes and has some strength; I am no longer weak and have power. This means that the medication I am taking now at least gives me strength and does its job well in my body.’ (Participant A, Male, 36 years)

‘I mean, I never had any side effects that they said I would have or maybe make me sick. They said that sometimes the pills can make you ill at the beginning of your treatment, but I never experienced any of those things. According to me, I see a change that is good since I started taking the treatment … I see myself having more energy and eating more, so I assume that it’s the new pills that do that to me.’ (Participant K, Male, 37 years)

‘Eish, they are fine. When I take them every day, my body becomes fine. I eat a lot sometimes, and I can do many things. [*pause*] … Maybe I can jog a bit for a distance and build strength. I also gained weight; that’s how I see the treatment is suitable.’ (Participant E, Female, 28 years)

**Sub-theme 1.2: persistent adherence to new treatment dolutegravir:** The findings revealed that participants showed persistent adherence to a new treatment, and it was echoed that one main reason for that was the treatment being tolerable:

‘You have to take them and not skip days because the more you skip, the more the virus has power, and … what is it again? Your immune system starts to decrease. So, you have to take them every day and should take your treatment accordingly. I take mine every day at eight at night, and the fact that the treatment does not give me problems makes it easy for me to take it.’ (Participant E, Female, 28 years)

‘I can say that, for the treatment to work effectively, you need to take it accordingly and don’t skip your treatment. If you don’t do so, you won’t see its effectiveness. It is essential to listen to the instructions given at the clinic. When they say take your treatment in the morning or at night, do as you are told, and then you will be fine. It would be best to do something to remind you to remember the time to take your treatment. It’s a good thing the treatment is okay in my body, so I have no reason not to take it, and I make sure to take mine at 7 o’clock every night.’ (Participant L, Female, 33 years)

Most participants showed great acceptance of the new treatment, dolutegravir and acknowledged the benefits that the treatment had in their lives. Adherence to the new treatment was also an indication of acceptance of the treatment, as it was shown to be tolerable by most patients.

#### Theme 2: Level of knowledge regarding the new treatment (dolutegravir)

The level of knowledge regarding the new treatment, dolutegravir, emerged as the second theme and addressed two sub-themes: insight into the importance of taking the new treatment, dolutegravir and lack of knowledge regarding the new treatment.

**Sub-theme 2.1: Insight into the importance of taking new treatment:** In most cases, participants reported being aware of the importance of effectively taking their treatment to control the HIV virus:

‘Yeah, I don’t know how to explain it. Let me say that when you take treatment, at first, it will try to adjust with your body to kill the virus that is in your body, but it does not mean that you are cured when you drink them; they just manage the disease by making the virus inactive can make you sick. But if you take your medication correctly, it means that these pills can kill this disease, and you can be cured.’ (Participant B, Female, 41 years)

‘You have to take them and not skip days because the more you skip, the more the virus has power, and … what is it again? Your immune system starts to decrease. So, you have to take them every day and should take your treatment accordingly. I take mine every day at eight at night, and the fact that the treatment does not give me problems makes it easy for me to take it.’ (Participant E, Female, 28 years)

‘The new treatment should be taken every day so that the HIV disease can be managed and not make you sick; then you will live normally. But if you don’t take it accordingly, you will get sick, and everyone will see that you are ill, and now and then, you won’t be able to hide the fact that you are sick. Taking treatment gives you a chance to live like any average person and work any job you want, just like other people.’ (Participant L, Female, 33 years)

**Sub-theme 2.2: Lack of knowledge regarding the new treatment (dolutegravir):** The majority of participants showed a lack of knowledge about the new treatment. Additionally, it was evident that they were unaware that their previous regimen had changed to the new one. This was expressed as follows:

‘Aii, I’m not sure … [*laughs*]. They give us different containers every time, so I’m unsure if it’s still the same treatment. The inside is the same; it’s just that the package is different, so I’m not sure what that means … They tend to change the bottles. Today, you get a blue bottle; next month, it’s a white bottle, and then, the other month, you can get a blue one again.’ (Participant K, Male, 37 years)

‘I don’t have much perception of my treatment. What I know is that you must take your course accordingly … I mean, you should take the treatment the way the nurses told you. You should not skip taking your medication and adhere to your dates for collection of treatment. Now I feel like I am fine, I am 100 per cent fine … Yes, they changed mine, but I did not ask why they changed them. In my mind, I thought it was just staged. You have completed the first stage and are now in the second stage.’ (Participant I, Female, 35 years)

‘What I know about this treatment … I don’t have anything I know; I take the treatment with the perception that, since I was sick and didn’t know who I was, they said I must take the treatment every day, and that was it.’ (Participant J, Female, 30 years)

‘I know that the treatment is to heal us, and we should take it every time. If you take them correctly, you won’t have a problem; you can live like anyone else.’ (Participant G, Male, 40 years)

Overall, participants seemed to know the importance of taking treatment, but the actual insight into the new treatment, dolutegravir, was deficient in most of them.

#### Theme 3: The burden of taking the previous regimen

The findings showed that the majority of participants started their HIV treatment journey with a different ART regimen and were switched to DTG-based regimen. A few participants had DTG-based regimen as their first and only regimen. A theme named the burden of taking ART treatment emerged with its sub-themes, namely the side effects of the previous regimen and the side effects of DTG-based regimen.

**Sub-theme 3.1: Side effects of the previous regimen:** Most of the participants interviewed were experienced-positive patients who started their HIV treatment journey on a different ART regimen and then were switched to a DTG-based regimen. While comparing the new treatment to their previous regimen, a common concern expressed was having experienced unpleasant side effects in their previous regimen, which made it difficult for them to tolerate the treatment. Participants in the following quotes echoed this sentiment:

‘In the beginning, when I was taking the HIV treatment I was taking before the new ones, they treated me somehow. I wondered if I should stop taking them or go to the clinic and report. When I was asleep at night, I would feel dizzy while in bed; I would dream of weird things. I would dream of people who died. I would see scary things as if I were falling underneath the bed. So, as I was explaining to them, they told me that these pills are like this. They are fighting the disease in my body.’ (Participant B, Female, 28 years)

‘When I started taking medication for HIV, I was taking a different kind of HIV treatment. I’m just unsure of their name, but it was orange-ish. Those pills, Eeeh, they were powerful. They would make me have scary dreams, but with time, when I changed to these pills that I am now taking, I am no longer experiencing those scary dreams. You understand. You also find my body itching, but with these new pills, my body does not itch.’ (Participant A, Male, 36 years)

‘When I started taking my first HIV treatment, I would feel burning here [*points at stomach*] … I would feel some dizziness, but they told me that after 6 days, I would feel better, and I should take treatment at night. Then, after 6 days, they changed me from taking the treatment at night, and I started taking it in the morning. I did have dizziness and a feeling like I wanted to vomit, but then I was okay. As time goes by, they change containers, and I don’t know if it is still the same treatment. They would take blood every February and then say it’s all right.’ (Participant H, Male, 45 years)

**Sub-theme 3.2: Side effects of DTG-based regimen:** A small number of those interviewed started their HIV treatment journey on a DTG-based regimen and stated that they had experienced side effects of DTG:

‘No, it has always been there, the new treatment. Initially, I would feel dizzy when taking the treatment. Sometimes, I would feel like vomiting, but never vomited, and I would take water and sit down to relax. Then, after 10 min to 15 min, I would feel better until they adjusted to my body. Now I am fine.’ (Participant E, Female, 28 years)

‘Not really; at first, when I started taking the new treatment, I was having problems with sleep. I was very energetic, making it hard to sleep at night. I would be up until 2 am; that was the only thing. But they helped me with the tiredness. I am more energetic now because of the pills, and I am back at work and working well. I don’t have a problem. I can live like any person.’ (Participant L, Female, 33 years)

The results suggested that most interviewees had experienced side effects on their previous regimen. The experienced patients expressed negative comments about their previous regimen. A relatively small number of participants who started their treatment with only DTG-based regimen did not experience side effects from the treatment at the early stages of starting treatment.

#### Theme 4: Reason for non-adherence to dolutegravir

Non-adherence to a DTG-based regimen was revealed by a few participants and emerged as the theme with sub-themes, namely alcohol use issues and work-related issues.

**Sub-theme 4.1: Alcohol use and work-related issues:** It was identified that alcohol use was a factor that made a few participants not comply with their treatment. This was echoed as follows:

‘Eeeh, mostly when I skip, to be honest with you, let me not hide anything. Honestly, I skip due to alcohol. So, when I’m at a drinking place, I can’t take a pill and put it in my pocket. The reason why the pills have to remain in their container is so that they are not exposed to air. So, if I take it and put it in my pocket, it will be exposed to air and will not work as effectively as it would have if it were in a container.’ (Participant A, Male, 36 years)

The same participant echoed that work-related issues often affect his time taking medication:

‘If I receive a sudden call that I am needed urgently, maybe by family or a job, since I am job-hunting, they say I have an interview. When you get to Marble Hall, you take more time than expected and get transport home late. We are drinking treatment at a specific and same time, and I drink mine at eight at night. So, I would sometimes return home after 8 or 9. According to what I told myself, if I miss the time to take my treatment, then I won’t take it until the following day.’ (Participant A, Male, 36 years)

Another participant said:

‘I work as a taxi driver and do long-distance trips from Marble Hall to Pretoria. Sometimes, I will go to Pretoria and come back this side late, and it will be past my time to take treatment. I usually take my treatment at 8 pm. when I am home, and when I go to work, I don’t carry it with me because I know I am coming back. I am trying to make sure I am home before the time to take my treatment.’ (Participant E, Female, 28 years)

In summary, the findings on theme 4 revealed non-adherence to treatment from a few participants. It was further outlined that reasons for non-adherence were patient factors rather than side effects of the treatment.

## Discussion

This study explored the perceptions of the DTG-based regimen among PLHIV in Limpopo province. The findings revealed that they were compelling, demonstrating that most participants positively accepted the new treatment, dolutegravir. These perceptions align with existing literature that highlights a high degree of acceptability of dolutegravir among patients (Abudiore et al. 2023; Zakumumpa et al. 2021). Improved appetite and subsequent weight gain were frequently reported advantages. This observation was consistent with studies that reported increased body weight among patients transitioning to DTG-based regimens (Hickey et al. 2023; Thivalapill et al. 2021). While weight gain may be beneficial for individuals who struggle to gain weight, it may be a concern for those at risk of obesity. With that said, healthcare providers must advocate for a healthy lifestyle incorporating regular physical activity and a well-balanced diet to address potential health risks.

Participants also associated DTG-based regimen use with improved physical strength, enabling them to exercise and perform their work duties without concerns and maintain productivity. While literature directly supporting this finding is limited, it suggests an emerging area of interest that warrants further investigation. This discovery could provide insights into the pharmacodynamics of DTG use and its potential role in enhancing physical strength.

A few participants attributed their ability to give birth to HIV-free children to the DTG-based regimen, reflecting its well-documented capacity for rapid viral suppression. This finding resonates with the findings from several studies that reported a rapid decrease in viral load to less than 50 copies on a dolutegravir-based regimen, compared to a reduction with an EFV-based regimen (Kawuma 2023; Lockman et al. 2021). Such perceptions illustrate how patients interpret treatment effectiveness in tangible, life-affirming ways that extend beyond clinical outcomes.

In addition to recognising its benefits, participants reported strong adherence to the DTG-based regimen, largely because of its tolerability and minimal side effects. The study established consistent adherence to treatment among participants. The high level of tolerability associated with the DTG-based regimen ultimately enhances individual adherence, which, in turn, provides reassurance to the healthcare system by reducing the likelihood of drug resistance. These findings mirror those of other studies that reported over 50% adherence among patients in the dolutegravir group compared to non-dolutegravir groups (Cardoso et al. 2019; McCluskey et al. 2021). In addition, a study by McCluskey et al. argued that participants better tolerated dolutegravir-containing regimens than EFV regimens (McCluskey et al. 2021). The findings are positive, as they indicate that patients have a deep comprehension of adhering to treatment, which suggests a higher likelihood of maintaining viral suppression over time. Such insights enhance the hope of reaching the international objective of eliminating AIDS, as awareness of adherence and personal responsibility among patients are crucial factors in achieving effective long-term management of HIV.

Most participants highlighted the importance of taking dolutegravir at a specific time each day to guarantee its effectiveness. Despite this awareness, participants revealed limited knowledge about DTG itself and were often uninformed about their current treatment regimen. Many reported being unaware of the reasons for switching regimens and relied heavily on healthcare providers’ authority. They knew that their prior regimen had been changed but did not comprehend the clinical reasons behind the switch of regimen. Some acknowledged that they never sought more information from the healthcare providers. This knowledge gap, which has also been noted in other emerging studies, highlights the need for improved patient education and continuous professional development among healthcare providers (Hussen Tale, Tegegne & Belay 2023; Ngowi et al. 2022). This shortfall may also be compounded by healthcare providers’ limited confidence in discussing a relatively new drug, which in turn restricts the depth of information patients receive. Kwame and Petrucka (2020) stress the importance of enhancing education for healthcare providers to guarantee that patients receive proper information and appropriate support. Without this, patients are at risk of being misinformed, having diminished trust and lacking control in making informed choices about their treatment. This research highlights the critical necessity of improving patient education and training for healthcare providers regarding the DTG-based regimen.

Participants also compared the DTG-based regimen with their previous regimens, as most had previously been initiated on another type of ART regimen before transitioning to a dolutegravir-based treatment. The majority were previously on the EFV-based regimen, which was often associated with adverse effects such as nightmares, dizziness and rash. These findings align with several studies that have documented the central nervous system (CNS) side effects of EFV (Muche, Kiflu & Ayalew 2020; Nelson et al. 2021). Dermatological effects, such as rash, were also commonly reported (Singh et al. 2023). Such side effects often compromise treatment adherence and quality of life and in some cases contribute to early discontinuation of EFV-based regimens.

While the dolutegravir-based regimen is currently considered the preferred option for both first- and second-line ART because of its efficacy, tolerability and high barrier to resistance, EFV has not been completely phased out. The WHO still recommends EFV as part of the first-line regimen, particularly in contexts where dolutegravir is not available, contraindicated or when patients remain stable and tolerate EFV well. Therefore, delaying the switch to the DTG-based regimen may be reasonable in cases where patients show sustained viral suppression and experience minimal side effects with EFV.

Transitioning to the DTG-based regimen was perceived as a relief, improving both adherence and quality of life. Some participants who had only experienced a DTG-based regimen reported mild side effects, including dizziness, vomiting and insomnia. Similar reports in the literature continue to fuel debate about DTG’s tolerability in real-world settings and indicate that there are cases of DTG-based regimen discontinuity among PLHIV because of neuropsychological side effects, such as low mood or depression and insomnia (Chanie et al. 2025; Perez-Valero et al. 2023). Given the findings of this study, ongoing monitoring by the nurses and clinicians remains crucial to ensure early identification and management of these side effects. While the side effects of DTG remain a topic of debate, studies have also demonstrated its high efficacy and robustness in suppressing HIV compared to other non-DTG regimens (Phillips et al. 2019; Evitt et al. 2023). Therefore, the benefits of dolutegravir outweigh its associated risks.

Work-related schedules and alcohol use also emerged as barriers to consistent adherence. Long work hours, commuting and social drinking contributed to occasional missed doses, echoing findings from previous studies linking alcohol use with non-adherence (Adrawa, Alege & Izudi 2020; Katjiuanjo 2023). While these challenges were anticipated, interestingly, participants did not report DTG treatment-related challenges as major obstacles but rather patient-related factors, with many noting that even when they experienced mild adverse effects from DTG, these resolved over time without discontinuation of treatment. Despite these challenges, participants demonstrated strong motivation to continue treatment, suggesting that DTG’s acceptability may mitigate some behavioural barriers commonly associated with ART non-adherence.

Overall, the findings affirm the DTG-based regimen’s acceptability and tolerability among PLHIV in this setting while underscoring the need for strengthened patient education and ongoing support from healthcare providers. By deepening understanding of patients’ lived experiences and perceptions, this study contributes to the growing body of evidence informing patient-centred HIV care and supports the continued rollout and management of DTG-based regimens for patients in South Africa.

### Limitations

This study had several limitations that should be considered when interpreting the findings. It was conducted in selected primary healthcare facilities within Limpopo province, Sekhukhune district, which may limit the generalisability of the results to other provinces or settings with different healthcare contexts. This study also relied on self-reported perceptions, which may be influenced by social desirability bias, as participants might have provided responses they believed were expected. As this is a qualitative study, the findings provide depth of understanding but cannot be used to establish causal relationships between dolutegravir use and patient outcomes.

### Recommendations

The following are recommendations from this study.

For practice:

- To enhance health education initiatives to improve patient awareness and understanding of dolutegravir, as a lack of knowledge has been observed despite the acknowledged advantages.
- To provide continuous in-service training to empower healthcare workers to ensure they remain updated with the new guidelines and HIV management, with the aim of providing adequate health education to patients.
- Healthcare providers should also include regular counselling sessions to discuss patient-related factors that might lead to non-adherence.

For future research:

- Further research should be carried out to investigate the root causes of non-adherence and to assess specific strategies designed to improve treatment results in various environments.

## Conclusion

The study aimed to explore and describe patients’ perceptions of dolutegravir in Limpopo province. The results showed a strong level of acceptance among participants, who highlighted perceived advantages like enhanced appetite and greater physical strength. Dolutegravir was mostly well accepted, which had a positive effect on adherence. The side effects that were reported were minor and did not lead to stopping the treatment, which helped enhance overall satisfaction with the regimen.

Despite these positive experiences, the study revealed a striking lack of awareness regarding dolutegravir among patients. This highlights the need for targeted health education to strengthen knowledge and empower patients in managing their treatment. Compared to previous regimens, dolutegravir was viewed more favourably because of its improved tolerability and fewer adverse effects. Nonetheless, the DTG side effects did not result in treatment discontinuation.

As one of the pioneering studies conducted in Limpopo province, this research offers significant insights into patient experiences with dolutegravir. However, it also identified a certain level of non-adherence to the dolutegravir regimen, primarily associated with patient-related factors. Addressing these barriers in a timely manner is pivotal and requires further research and the development of tailored interventions. Such studies are of great importance to enhance treatment outcomes and contribute to the global goal of ending AIDS by 2030.
